# Current Therapeutic Advances Targeting EGFR and EGFRvIII in Glioblastoma

**DOI:** 10.3389/fonc.2015.00005

**Published:** 2015-01-29

**Authors:** Emily Padfield, Hayley P. Ellis, Kathreena M. Kurian

**Affiliations:** ^1^University of Bristol, Bristol, UK; ^2^Brain Tumour Research Group, Institute of Clinical Neurosciences, University of Bristol, Bristol, UK

**Keywords:** EGFR, EGFRvIII, EFGR inhibitors, glioblastoma multiforme, molecular marker

## Abstract

Epidermal growth factor receptor (EGFR) and EGFRvIII analysis is of current interest in glioblastoma – the most common malignant primary CNS tumor, because of new EGFRvIII vaccine trials underway. EGFR activation in glioblastoma promotes cellular proliferation via activation of MAPK and PI3K–Akt pathways, and EGFRvIII is the most common variant, leading to constitutively active EGFR. This review explains EGFR and EGFRvIII signaling in GBM; describes targeted therapy approaches to date including tyrosine kinase inhibitor, antibody-based therapies, vaccines and pre-clinical RNA-based therapies, and discusses the difficulties encountered with these approaches including pathway redundancy and intratumoral heterogeneity.

## Introduction

There is an urgent need for new molecular targeted therapies for newly diagnosed GBM (1–4). Recent data from The Cancer Genome Atlas project has proposed various subtypes of GBM, each with distinct molecular properties and genetic aberrations (5), although there is increasing recognition that there is molecular heterogeneity within individual tumors (6–8). Primary GBM is frequently associated with epidermal growth factor receptor (EGFR) amplifications compared with secondary GBM, which may arise from lower grade precursors (5, 9).

**Figure 1:**
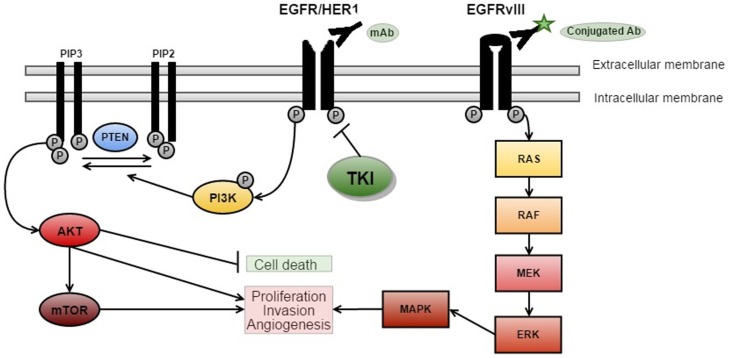
**EGFR signaling and targeted therapies**. There are three key signaling pathways in GBM. The RTK/RAS/PI(3)K pathway is involved in cell growth, apoptosis resistance, invasion, and migration (pictured above with targeted therapies). The other critically important pathways that regulate cell proliferation and survival are p53 and RB signaling (5) [adapted from Ref. (14)].

Overall, aberrant amplification, deletion, or mutation of at least one receptor tyrosine kinase (RTK) has been found in 67.3% of GBM, with EGFR accounting for 57.4% (10) (as shown in Figure 1). In addition, around 50% of patients with EGFR amplification harbor a specific mutation – known as EGFRvIII – which results from an in-frame deletion of exons 2–7 (11, 12). EGFRvIII can also be present independently of EGFR amplification (13).

Due to the frequency of EGFR aberrations, many EGFR-targeted therapies are in development or clinical trials (14). Although EGFR kinase inhibitor therapy has shown initial success in other cancers such non-small cell lung cancer (15), previous trials in glioblastoma have been unsuccessful to date (16–18). More recently, interest has focused on an anti-EGFRvIII vaccine (known as rindopepimut), which has entered clinical trials (19).

## EGFR Signaling in GBM

Epidermal growth factor receptor signaling has an important role in many cancers, as cellular proliferation is mainly controlled by growth factors and their receptors (Figure 1). EGFR (also known as HER1 or ERBB1) is a receptor belonging to the ERBB family of the RTKs (20). Ligand-binding by EGF results in the activation of the RTK/RAS/PI(3)K pathway (21) via receptor phosphorylation, ultimately resulting in cellular proliferation, angiogenesis, and increased local tissue invasion as well as resistance to apoptosis (21–23).

Ligand-binding to RTKs simultaneously activates PI3K (see Figure 1) (22). PI3K phosphorylates phosphatidylinositol 4,5-biphosphate (PIP2) to phosphatidylinositol 3,4,5-triphosphate (PIP3), which results in further activation of AKT (22). AKT then promotes activation of mTOR – a protein, which exists as two complexes; mTORC1 and mTORC2 (24). Activation of mTORC1 promotes cellular growth by biosynthesis of proteins, lipids, and organelles in addition to inhibition of catabolic activity (22). mTORC2 activation results in phosphorylation and subsequent activation of several molecules (including AKT) that are involved in cell survival, metabolism, and proliferation (22). PTEN is a major inhibitor of this pathway by preventing phosphorylation of PIP2 to PIP3 (22). Loss of PTEN, such as the homozygous deletions observed in around 36% of gliomas, results in dramatic up-regulation of this pathway, and may be a major source of resistance to EGFR therapies (5, 22, 23).

*In vitro* studies suggest that the signaling mechanism of EGFRvIII cells can confer resistance to EGFR inhibitors (25), because EGFRvIII signals via an mTOR2 pathway whereas wtEGFR uses mTOR1 (24).

## EGFRvIII Signaling

EGFRvIII is often co-expressed with wtEGFR, typically in tumors with EGFR over-expression, which complicates our understanding of its contribution to tumorigenesis (13, 26, 27). It has been suggested that the transformation happens intracellularly, with EGFRvIII being phosphorylated in an EGFR-dependent manner leading to increased downstream STAT signaling (27). There is also evidence in GBM of EGFRvIII internalization to form an oncogenic complex with STAT3 (27).

A retrospective analysis of clinical trials found that of 40 patients with *EGFR* amplification, those also expressing EGFRvIII had significantly shorter survival (0.839 years) than patients without (1.374 years), *P* = 0.0031 (13). Considerable growth advantage has been observed in EGFRvIII transfected GBM cell lines (U87MG.EGFRvIII), when compared to wtEGFR cell lines (28–30). This growth advantage is thought to result from an elevated proliferation rate coupled with a reduction in apoptosis (28). Similar to EGFR signaling, EGFRvIII activates the RTK/RAS/PI3K pathway as a result of EGFRvIII expression (31, 32). This results in increased levels of phosphorylated AKT and reduced levels of P27^KIP1^, a cell cycle regulator that inhibits G_1_–S phase transition in cell lines (33). Furthermore, abnormal spindle-like microcephaly-associated (ASPM) protein expression has been described in the U87MG.EGFRvIII cells compared to parental U87MG cells (34). ASPM promotes neural stem cell self-proliferation and hence it has been postulated the increased expression results in enhanced GBM progression by promoting cancer stem cell self-renewal (34).

The proliferative effects of EGFRvIII may be potentiated by the anti-apoptotic nature of brain tissue through up-regulation of Bcl-X_L,_ which is a potent inhibitor of apoptosis (35). EGFRvIII has also been shown to have a role in tumor invasiveness, for example, *in vitro* studies demonstrated that U87MG.EGFRvIII cells displayed up-regulation of genes that promote an invasive phenotype such as matrix metalloproteinase (MMP)-13 (36). In both scratch tests and Matrigel Invasion Chamber assays, the cells also showed greater ability for migration and local tissue invasion than wtEGFR cells (36).

In the presence of amplified EGFR, it has been found that regulation of the nuclear factor kappa-light-chain-enhancer of activated B cells (NF-κB) pathway through IκBαM gene transfer could play a role in glioma angiogenesis by regulating the expression of vascular endothelial growth factor (VEGF) and interleukin-8 (IL-8) (37).

## EGFR-Targeted Therapies

Four modes of targeted therapies have been used to target EGFR including tyrosine kinase inhibitors (TKIs), antibody-based therapy, immunotherapy, and pre-clinical trials of RNA therapies. TKIs are small-molecule inhibitors, which bind to the ligand-binding site on the extracellular domain, and are the most clinically advanced EGFR-targeting therapy to date (14). Antibody-based therapy uses monoclonal antibodies that correspond to the receptor landscape to inhibit signaling, and can also use conjugated antibodies that allow toxins or radioactive isotopes to be targeted to specific cells (38). The current immunotherapy for EGFRvIII can be administered in the form of an intradermal vaccine CDX-110 and granulocyte macrophage-colony stimulating factor (GM-CSF) (39). RNA therapies will also be discussed, which involve creating antisense oligonucleotides or siRNA complementary to the regions that it would be clinically beneficial to silence (40).

## Tyrosine Kinase Inhibitors Targeting EGFR

Epidermal growth factor receptor TKIs gefitinib and erlotinib (see Table 1) have been found to significantly increase progression-free survival in non-small cell lung carcinoma (NSCLC) patients, with one meta-analysis reporting 42.9% of patients receiving TKI therapy reaching at least 1 year of progression-free survival compared to 9.7% with chemotherapy (41). A study of gefitinib as palliative therapy for patients with brain metastases from NSCLC found that 45% of patients experienced symptom improvement, with the experimental group maintaining progression-free survival for 6 months longer than the control group (42). As an initial therapy for asymptomatic brain metastases in never-smokers with adenocarcinoma of the lung, the combination of gefitinib and erlotinib has shown response rates of up to 70% (43). Lapatanib is another tyrosine kinase inhibitor used in treatment of HER2^+^ breast cancer, which when used in combination with capecitabine was found to increase progression-free survival to 8.4 months compared to 4.4 months receiving capecitabine monotherapy at the primary endpoint of a clinical trial of metastatic breast cancer patients (44). In the monotherapy group, 11 patients had CNS metastases compared with 4 in the combination therapy group (44), though lapatinib has not yet been shown to have activity against recurrent GBM in clinical trials (45).

**Table 1:** **A summary of therapies targeting EGFR and EGFRvIII**.

Therapy	Target	Current clinical applications	Problems reported in glioma trials	Reference
**Monoclonal antibodies**
Cetuximab (L01XC06)	EGFR/HER1	Colorectal cancer		
		Head and neck cancer		
Panitumumab (L01XC08)	EGFR/HER1	Metastatic colorectal cancer	Crossing BBB	
Nimotuzumab (L01XC)	EGFR/HER1	Squamous cell carcinoma of head and neck	Hypersensitivity	(36, 46–48)
		Orphan status for glioma and pancreatic cancer	Nervous system toxicity	
125 I-Mab 425	EGFR	N/A		
mAb806	EGFRvIII	N/A		
DAB389EGF	EGFR	N/A		
**Small molecule inhibitors**
Gefitinib (L01XE02)	EGFR/HER1	NSCLC		
Erlotinib (L01XE03)	EGFR/HER1	NSCLC and pancreatic cancer	Insufficient delivery	(14–17, 39–43, 49)
Lapatinib (L01XE07)	EGFR/HER1/HER2	HER2^+^ breast cancer	Resistance to inhibition	
Afatinib (L01XE13)	EGFR/HER1/HER2/HER4	Metastatic NSCLC		
Dacomitinib	EGFR/HER1/HER2/HER4	N/A		
**Vaccines**
Rindopepimut (CDX-110)	EGFRvIII	N/A	Tumor heterogeneity	(37, 50–52)
			Patient selection	

Pre-clinical results demonstrate the ability of TKIs to inhibit tumor cell growth, angiogenesis, survival, and proliferation in several different EGFR transfected GBM cell lines (36, 49, 53, 54). However, these results do not appear to be clinically translatable, as response rates in GBM patients are disappointing for many inhibitors including gefitinib and erlotinib (55, 56). One explanation of this could be that TKIs are most efficacious when targeting tumor cells that express mutations in exons 19 and 21 of the EGFR kinase domain, which has been identified in various cancer types but has not yet been elucidated in GBM (14).

## Antibody Targeting of EGFR

Despite the success of antibody-based therapy in the treatment of renal cell carcinoma, melanoma, and hematologic cancers, these results have not been replicated in GBM (46, 57, 58). Conjugated and unconjugated antibodies have been developed to target both wtEGFR and EGFRvIII, the most successful so far being cetuximab, panitumumab, and nimotuzumab (41, 47). The unconjugated antibodies bind the extracellular domain of EGFR, and they are also suggested to cause internalization of EGFRvIII, though clinical trials have had varying results (48).

Treatment of EGFR-amplified GBM cells with cetuximab in subcutaneous and intracranial mouse xenografts has been found to result in a significant decrease in proliferation, and an increase in overall survival as well as apoptosis (59). A decrease in the expression of VEGF in cell supernatant was observed using an enzyme-linked immunosorbent assay, suggesting further potential for application in GBM, as this signaling pathway also contributes to tumor maintenance and angiogenesis (59).

A Phase II study stratified patients depending on their EGFR gene amplification status and both groups were administered cetuximab intravenously (60). Cetuximab had little effect in both study groups and the median overall survival was 5 months, eliciting no significant correlation between EGFR status and response or overall survival (60). Other clinical trials involving similar antibody-based therapies have been equally unsuccessful, though a decrease in skin toxicity has been reported with use of nimotuzumab, which could increase its viability as an adjuvant therapy in GBM (47).

In a Phase III study, patients were administered nimotuzumab with concurrent radiotherapy (47). Although there was no statistically significant difference in overall survival of patients, the patients with the greatest median overall survival had molecular markers of EGFR amplification and unmethylated MGMT (47).

Antibodies, which utilize toxins or radioisotopes, could provide a potent adjuvant therapy for GBM as they enhance cell killing by the immune system in addition to inhibition of EGFR signaling (61). Various early clinical trials report that administering the radiolabeled antibody ^125^I-MAb 425 intravenously, either alone or with standard of care treatment, significantly improves median survival (38, 50, 61). In the largest Phase II trial to date combined treatment of ^125^I-mAb 425 and TMZ provided the greatest survival benefit with a median survival of 20.4 months, compared to treatment of ^125^I-mAb 425 alone, which was 14.5 months (50). Antibodies conjugated to death receptor agonists have been reported to induce apoptosis in GBM cell lines, for example, the scFvM58–sTRAIL fusion, which has been shown to selectively target GBM cells that express multidrug resistance protein 3 (62).

## Immune Therapy Using Vaccines

In initial Phase I trials, vaccinations comprising dendritic cells (DCs) primed with EGFRvIII peptides were found to be safe, with only grade I and II skin reactions at the vaccine injection site reported (51). Patients were also found to be immunologically responsive when their cellular immune responses were tested regularly using skin tests (51). Histological analysis in recurrent GBM patients who received the same vaccine showed no residual expression of EGFRvIII, demonstrating that the vaccine can effectively eliminate EGFRvIII cells, though all other cell types remain intact (63).

The Phase II trial “ACTIVATE” included 19 patients with newly diagnosed GBM received vaccines comprising PEPvIII-KLH (the EGFRvIII peptide coupled keyhole limpet hemocyanin to illicit both humoral and cellular immune responses) and GM-CSF (64). Progression-free survival was 12 months and patients who demonstrated immune sensitization to EGFRvIII had an overall survival of 47.7 months in comparison to 22.8 months for those who did not (64). It is important to note that positive results in these clinical trials could be due at least in part to the use of GM-CSF, as its use in cancer immunotherapy is enhancement of immunotherapeutic mechanisms of tumor destruction (52).

Another arm of this trial “ACT II” compares rindopepimut/GM-CSF concurrently with either standard or dose-intensive adjuvant TMZ (19). All patients were found to have an immune response to EGFRvIII; however, patients in the dose-intensive cohort had an even greater serum response, which may be partly due to a decrease in regulatory T cells (19). Most importantly, overall survival was greatly improved (23.6 months) in comparison to historical case-matched controls (19).

The larger “ACT III” trial sought to evaluate the clinical efficacy of the peptide vaccine CDX-110 in addition to radiotherapy and TMZ and produced similar results (65). The study was initially a randomized Phase II/III study, but patients belonging to the non-vaccine group withdrew from the trial (66). The median OS was 24.6 months compared to 15.2 months for matched EGFRvIII-positive controls (66). In addition, the results appeared to show a benefit in patients with methylated and unmethylated MGMT promoters (66).

A Phase III clinical trial, “ACT IV,” which compares rindopepimut plus GM-CSF and TMZ to current standard of care alone with a control (keyhole limpet hemocyanin) has been undertaken. Screening closed for this study on 30/9/14 with around 700 patients enrolled, and primary data collection is expected to take place in late 2016 (NCT01480479). A further Phase II trial (ReACT) is also underway involving patients with recurring EGFR-positive GBM receiving the EGFRvIII vaccine in addition to bevacizumab (NCT01498328). Also, at Stanford University a Phase I trial is underway utilizing the EGFRvIII vaccine in children with diffuse intrinsic pontine gliomas, as EGFR expression has been found to occur in ~50% of the tumors studied (67).

## Pre-Clinical and Clinical Trials of RNA-Based Therapies

The use of antisense oligonucleotides to inhibit translation of mRNA has already yielded good results in pre-clinical studies for NSCLC and prostate cancer (68, 69). Following this, several experimental RNA methods targeting EGFR and EGFRvIII have been developed, including antisense oligonucleotides, RNA interference (RNAi), and ribozymes (40, 70, 71).

Antisense RNA appears to be efficacious in targeting EGFR expressing cells *in vitro* (70, 72, 73). Injection of vectors containing antisense RNA to target EGFRvIII into intracranial glioblastoma xenografts were found to reduce tumor volume by >40-fold compared with controls (74). In addition, in a U251 subcutaneous mouse model treated with antisense RNA and siRNA had significantly smaller tumor volumes by 29 and 19%, respectively, when compared to controls, further demonstrating efficacy *in vivo* (40).

Therapy with siRNA leads to post-transcriptional gene silencing that results in the destruction of the target mRNA (75). siRNA against EGFR has caused up to 90% knockdown of EGFR mRNA in U251 glioma cells (40). These results were reproduced using an intracranial xenograft mouse model, where median overall survival increased by almost 90% (40).

In pre-clinical studies, ribozymes targeting EGFRvIII were shown to inhibit ERM5–1 and U87MG glioblastoma cells (71, 76). In U87MG.EGFRvIII cells, anti-EGFRvIII hairpin ribozymes resulted in >90% reduction of EGFRvIII mRNA and a reduction in proliferation (71).

There may also be future potential for adjuvant miRNA-based therapies, as miR-7 has been shown to be an efficacious inhibitor of the EGFR signaling pathway in glioblastoma cell lines *in vitro* by direct inhibition of the EGFR receptor and further independent down-regulation of AKT, leading to a decrease in cell invasiveness (77). An increase in the radio-sensitivity of resistant cancer cells has also been described following miR-7 (78). The first miRNA-based cancer therapy (MRX34) has recently entered a Phase I clinical trial to evaluate its safety for use against primary liver cancer and liver metastases (NCT01829971). However, development of miRNA-based therapies against glioma may be considerably more difficult due to the lack of a delivery system sufficient to bypass the blood–brain barrier (79).

## Limitations of Targeted Therapies

Limitations using TKIs, such as erlotinib may have an inability to pass the BBB due to the presence of efflux transporters on the endothelial cells associated with the BBB (65). Additionally, at present very little is known about the long-term adverse effects of non-specifically inhibiting EGFR signaling using TKIs, and knowledge of the biological effects of EGFR inhibitors on GBM cells is still incomplete (14).

Drawbacks described using antibody therapy may relate to local compared with systemic administration, for example, in mouse models, systemic administration of an antibody directed to EGFRvIII had no improvement in survival compared to controls, but intratumoral injection of the antibody resulted in an increase of median overall survival of 286% (64). A further study in rats demonstrated that cetuximab applied by an osmotic mini-pump significantly reduced tumor growth in the brain versus systemic application – which failed to block tumor growth (52). This could be due to the large molecular weight of the antibodies reducing their ability to traverse the BBB without assisted transport vectors (80).

Cautious interpretation of the utility of the ACT IV immunotherapy trial is needed because patients eligible to enroll in the trial were very highly selected, for example, they were newly diagnosed with complete tumor resection and no evidence of progressive disease (NCT01480479). This is because it has been shown that in patients with tumor resection of <95% neither over-expression of wtEGFR nor the presence of EGFRvIII can be used independently to predict patient survival (81). One hypothesis is that a small tumor may still be relatively immunoprivileged; thus, the immune system may be relatively naïve to tumor antigens (39).

In RNA studies, AAV/shRNA vectors were found to be severely toxic, and caused fatality in 64% of mice due to oversaturation of RNAi pathways (82, 83). Furthermore, RNA entities are anionic, hydrophilic, and unable to enter cell by passive diffusion mechanisms, so the BBB is essentially impenetrable to any potentially therapeutic RNA molecules (84).

Moreover, intratumoral heterogeneity may be a complicating factor. A study cohort of 57 glioma cases, examining tumoral heterogeneity via immunohistochemistry found that in one case the over-expression and amplification were localized to one half of the glioma, with the other half demonstrating normal levels of EGFR expression (85). Furthermore, not all of the samples with genetic amplifications also had increased levels of RNA, which further complicates the assessment of the viability of using EGFR-targeted therapies (85).

## GBM Resistance to EGFR Inhibition

It has been previously shown that targeting of the EGFR receptor can lead to selection pressure for somatic mutations at other points in the pathway, such as inactivating phosphorylation of PTEN, leading to resistance to EGFR inhibitors (86), which could present a further problem for the ACT IV trial. Loss of PTEN has previously been found to be strongly correlated with treatment failure in GBM (87), to the extent that the analysis of EGFRvIII and PTEN levels may be used to predict tumor response to TKI therapy (88).

Additionally, in GBM there is a redundancy in activation of PI3K due to the availability of several types of tyrosine kinases upstream (86), including GFR1, MET, PDGFRα/β, and uPAR (89, 90). Increased activation of other members of the ERBB family of tyrosine kinases has also been described, as compensatory activation of ERBB2 and ERBB3 was noted after EGFR withdrawal in GBM cancer-stem-cell lines (91). This means that even after totally depleting EGFRvIII expressing populations of cells specifically, other tumor sub-clones with alternative mutations could be selected for, maintaining the tumor population and allowing functional resistance to EGFR-targeting therapies, and inhibitors of other ERBB family members may also be required for down-regulation of downstream elements (86, 91).

The loss of PTEN and increase in expression of other ERBB receptors may render EGFR signaling dispensable in the tumor, allowing growth and survival without EGFR signaling and thus negating the therapeutic viability of EGFR-targeting therapies in these cases (87, 88, 91). This uncoupling of the downstream pathways from EGFR signaling could be a possible explanation for the poor clinical responses exhibited with TKIs such as erlotinib (88).

Additionally, studies examining intratumoral heterogeneity found that it can be maintained by interactions between tumor cells, including the up-regulation of IL-6 production in EGFRvIII cells to activate neighboring wtEGFR cells, enhancing tumor growth and resistance to therapy (7). The mechanisms by which intratumoral heterogeneity arises are poorly understood; however, tumor cells have been found to reversibly increase or decrease levels of EGFRvIII expression in order to maximize their growth potential (92). Erlotinib resistance appears to be linked to EGFRvIII suppression in extrachromosomal DNA in order to successfully evade therapeutic mechanisms that target extrachromosomal oncogenes (92). Surprisingly, in this study, Nathanson *et al*. also described a reversal of erlotinib resistance within 72 h upon withdrawal of the drug, where extrachromosomal EGFRvIII DNA was dramatically upregulated, and restored sensitivity to TKI-induced cell death (92).

A study comparing the efficacy of lapatinib in lung cancer and GBM found that the lack of response to therapy in GBM could be due to the location of the mutation. Lung cancer EGFR mutations tend to occur in the kinase domain, whereas GBM EGFR mutations are mainly in the extracellular domain, which could allow the GBM mutant receptors sufficient flexibility within the kinase domain to accommodate lapatinib and other type II EGFR kinase inhibitors (93).

## Summary

The intratumoral heterogeneity of EGFR expression in GBM may ultimately limit the clinical utility of therapies such as TKIs, antibody-based therapies, and RNA-based therapies, because it will not be possible to target every single neoplastic cell in the tumor population. Moreover, it is likely that other sub-clones of tumor cells will arise or other pathways may be upregulated as a mechanism of resistance to EGFR-targeted therapies. Although problems with EGFR intratumoral heterogeneity and pathway redundancy will also apply to immunotherapy approaches, vaccines may ultimately be more attractive because they alert the immune system to the presence of tumor and may trigger a more non-specific tumoricidal immune response, which may potentially eradicate all tumor cells.

## Conflict of Interest Statement

The authors declare that the research was conducted in the absence of any commercial or financial relationships that could be construed as a potential conflict of interest.
